# Human genetics suggests differing causal pathways from HMGCR inhibition to coronary artery disease and type 2 diabetes

**DOI:** 10.1093/ije/dyaf223

**Published:** 2026-01-05

**Authors:** Seongwon Hwang, Ville Karhunen, Ashish Patel, Sam M Lockhart, Paul Carter, John C Whittaker, Stephen Burgess

**Affiliations:** MRC Biostatistics Unit, School of Clinical Medicine, University of Cambridge, Cambridge, United Kingdom; MRC Biostatistics Unit, School of Clinical Medicine, University of Cambridge, Cambridge, United Kingdom; MRC Biostatistics Unit, School of Clinical Medicine, University of Cambridge, Cambridge, United Kingdom; Wellcome-Wolfson Institute of Experimental Medicine, Queen’s University Belfast, Belfast, United Kingdom; Institute of Metabolic Science, University of Cambridge, Cambridge, United Kingdom; Department of Medicine, University of Cambridge, Cambridge, United Kingdom; MRC Biostatistics Unit, School of Clinical Medicine, University of Cambridge, Cambridge, United Kingdom; MRC Biostatistics Unit, School of Clinical Medicine, University of Cambridge, Cambridge, United Kingdom

**Keywords:** multivariable Mendelian randomization, colocalization, statins, drug target development, genetic epidemiology

## Abstract

**Background:**

Statins lower low-density lipoprotein cholesterol (LDL-C) and reduce the risk of coronary artery disease (CAD). However, they also increase the risk of type 2 diabetes (T2D).

**Methods:**

We consider genetic variants in the region of the *HMGCR* gene, which encodes the target of statins, and their associations with downstream consequences of statins. We use various statistical methods to identify causal pathways influencing CAD and T2D, and investigate whether these are the same or different for the two diseases.

**Results:**

Colocalization analyses indicate that LDL-C and body mass index (BMI) have distinct genetic predictors in this gene region, suggesting that they do not lie on the same causal pathway. Multivariable Mendelian randomization analyses restricted to variants in the *HMGCR* gene region revealed LDL-C and BMI as causal risk factors for CAD, and BMI as a causal risk factor for T2D, but not LDL-C. A Bayesian model averaging method prioritized BMI as the most likely causal risk factor for T2D, and LDL-C as the second most likely causal risk factor for CAD (behind ubiquinone). Colocalization analyses provided consistent evidence of LDL-C colocalizing with CAD, and BMI colocalizing with T2D; evidence was inconsistent for colocalization of LDL-C with T2D, and BMI with CAD.

**Conclusions:**

Our analyses suggest cardiovascular and metabolic consequences of statin usage are on different causal pathways, and hence could be influenced separately by targeted interventions. More broadly, our analysis workflow offers potential insights to identify pathway-specific causal risk factors that could provide possible repositioning or refinement opportunities for existing drug targets.

Key Messages

- We performed colocalization and cis-multivariable Mendelian randomization using genetic association data for variants in the *HMGCR* gene region to investigate causal pathways influencing coronary artery disease (CAD) and type 2 diabetes (T2D).
- Our analyses suggest that the impact of HMGCR inhibition on CAD risk is mediated by both low-density lipoprotein cholesterol (LDL-C) and body mass index (BMI), whereas for T2D, risk was mediated via BMI but not LDL-C.
- Our results suggest the possibility that targeted treatments could be developed to inhibit HMGCR in a more specific way that lowers CAD risk without increasing T2D risk.

## Introduction

Statins are a well-known class of medications that inhibit 3-hydroxy-3-methyl-glutaryl-coenzyme A reductase (HMGCR), the rate-controlling enzyme in the mevalonate pathway [1]. This is a metabolic pathway that synthesizes cholesterol and other organic chemicals. Statins are well-established by randomized clinical trials to lower low-density lipoprotein cholesterol (LDL-C) and reduce the risk of coronary artery disease (CAD), making them a cornerstone of cardiovascular disease preventive therapy [2]. Similar reductions in CAD risk have been observed for other LDL-C lowering agents [3–5]. However, statins have also been shown in trials [6] and population-based studies [7, 8] to increase risk of incident type 2 diabetes (T2D), and in some cases to worsen glycemic control [9] and progression to insulin requirement [10] in established diabetics. Despite this, statins provide an overall benefit with respect to microvascular and macrovascular cardiovascular disease [11], although a therapeutic option which isolates the LDL-C lowering effect of statins from its diabetogenic effects would be clinically desirable.

Genetic variants can be used to predict the results of clinical trials using a technique known as Mendelian randomization [12]. Individuals with certain genetic variants in the *HMGCR* gene region have a natural predisposition to increased inhibition of the mevalonate pathway that is analogous to taking a low-dose statin [13]. As genetic variants are inherited at random conditional on the parental genotype according to Mendel’s laws, epidemiological associations of variants in the *HMGCR* gene should reflect the downstream consequences of taking statins [14]. Empirical investigations have suggested that genetic associations in a well-mixed and homogeneous population are not systematically affected by confounding, and so the associations should be a reliable guide as to the effects of statins [15, 16]. Indeed, variants in the *HMGCR* gene region that associate with higher LDL-C are also associated with a greater risk of CAD [17] and lower risk of T2D [18], in line with clinical trials.

However, clinical trials of other LDL-C lowering agents (including PCSK9 inhibitors [3], NPC1L1 inhibitors [4], and bempedoic acid [5] have not demonstrated increases in T2D risk, and genetic associations of variants in corresponding gene regions show between-region heterogeneity in their associations with T2D risk [19, 20]. In contrast, genetic associations with CAD risk are proportional to the genetic associations with LDL-C for variants in different drug-mimicking gene regions [17, 21]. This suggests that LDL-C may not be driving the increases in T2D risk observed in statin trials.

The aim of this investigation is to explore the mechanisms linking the target of statins to CAD and T2D using multiple genetic variants in the *HMGCR* gene region. We use various statistical techniques to investigate causal pathways, including colocalization, multivariable Mendelian randomization, Bayesian model averaging, and Bayesian model selection. We discuss the implications of these analyses for lipid lowering treatment strategies in practice.

## Methods

### Study overview

**Figure 1.**
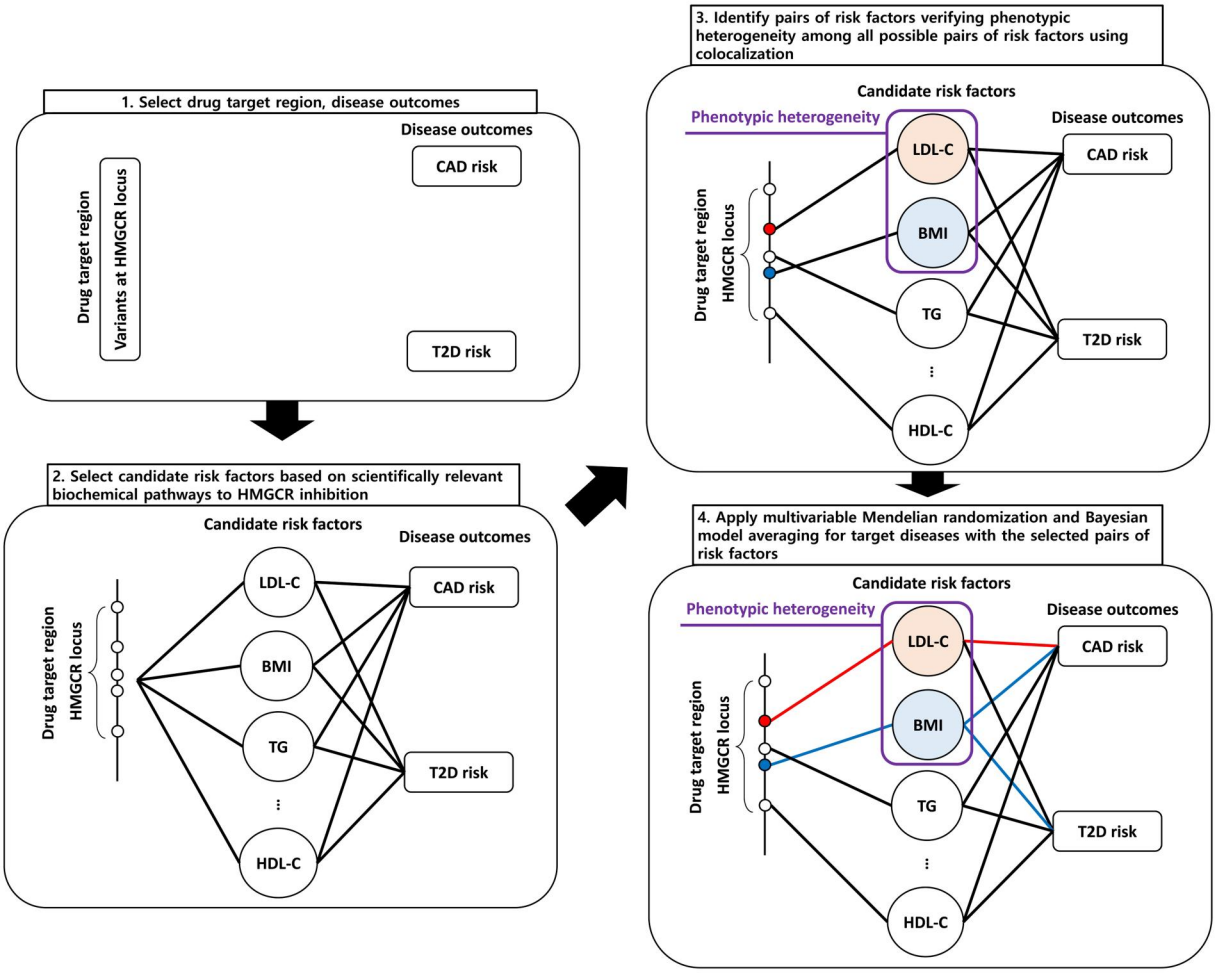
Overall flowchart of our workflow. Abbreviations: CAD: coronary artery disease, LDL-C: low-density lipoprotein cholesterol, HDL-C: high-density lipoprotein cholesterol, BMI: body mass index, TG: triglyceride, T2D: type 2 diabetes, *HMGCR*: 3-hydroxy-3-methyl-glutaryl-coenzyme A reductase.

We consider associations of genetic variants in the *HMGCR* gene region with risk factors that have been demonstrated to be consequences of statin usage, and with CAD and T2D risk. For each pair of risk factors, we perform colocalization to investigate whether the risk factor association signals are driven by the same or different variants [22]. For pairs of risk factors driven by different variants, we conduct multivariable Mendelian randomization analyses with CAD and T2D as outcomes [23]. We also use two Bayesian approaches to prioritize risk factors and hence identify the most statistically plausible risk factors affecting CAD and T2D: Bayesian model averaging to calculate the posterior probabilities of all possible risk factor models [24], and Bayesian variable selection to calculate the posterior inclusion probabilities of all risk factors [25]. While we would be cautious about an overly literalistic interpretation of the statistical results that we present here, strong differences in results for CAD versus T2D as the outcome would be indicative of different mechanisms by which *HMGCR*-regulated interventions on the mevalonate pathway may affect these two diseases. An overview of the analyses is displayed as Fig. 1.

### Risk factors

We selected 16 candidate risk factors based on scientifically relevant biochemical pathways to *HMGCR* inhibition and the availability of summarized genetic association data. These were: LDL-C, high-density lipoprotein cholesterol (HDL-C), triglyceride, sterol, ubiquinone, cortisol, testosterone, estradiol, aldosterone, 25-hydroxyvitamin D, bile acids, body mass index (BMI), leptin, acute insulin response, fasting insulin, and fasting glucose. This choice of variables provides comprehensive coverage of major lipids, steroid hormones (which are synthesized from cholesterol), and metabolic traits, as well as other cholesterol-derived compounds (25-hydroxyvitamin D and bile acids). Datasets used to obtain estimates of genetic associations with these risk factors are listed in Supplementary Table S1. All risk factor associations were derived in European ancestry participants only.

### Outcomes

Genetic associations with CAD were obtained from a large genome-wide association study comprising 181,522 cases and 984,168 controls predominantly of European ancestry [26]. Genetic associations with T2D were obtained from the Diabetes Meta-Analysis of Trans-Ethnic association studies (DIAMANTE) Consortium analysis of 80,154 cases and 853,816 controls of European ancestry [27].

### Statistical analyses

All analyses used genetic variants located within 10k base pairs of the *HMGCR* coding region (chromosome 5, positions 74,632,154–74,659,826 on build hg19). We consider a narrow range of variants around the coding region to ensure, as far as possible, that any genetic associations relate to inhibition of *HMGCR* and not to unrelated pathways.

Colocalization analyses were performed using coloc [28] and coloc-SuSiE [29] using default colocalization priors, prop-coloc-cond [30] with a pruning level of 0.4, and colocPropTest [31], and otherwise with default settings. When coloc-SuSiE finds multiple causal variants, we display results for the lead pair of credible sets in our plots and provide full results in a separate table. Where required, colocalization analyses used linkage disequilibrium matrices estimated in European ancestry UK Biobank participants.

We perform multivariable Mendelian randomization for pairs of risk factors that have strong evidence for having non-proportional genetic associations (i.e. evidence for non-colocalization). Multivariable Mendelian randomization analyses were implemented using the multivariable principal component generalized method of moments (MV-PC-GMM), which performs dimension reduction to reduce a large number of correlated variants to a manageable number of orthogonal principal components [32]. This method was chosen as pruning strategies for *cis*-Mendelian randomization can be inefficient, particularly for multivariable Mendelian randomization, as we require conditionally strong genetic predictors of each risk factor [33]. The method is implemented in the mr_mvpcgmm function in the MendelianRandomization package with the random effects option. We used a threshold of 99% of variance in the matrix of genetic associations with the exposure for selecting principal components, allowing for random-effects heterogeneity, and using variant correlation estimates obtained from 367,703 European ancestry UK Biobank participants. To assess the sensitivity of the results to the proportion of variance explained, we perform a sensitivity analysis to compare results with different numbers of principal components.

We regard this as a hypothesis-generating investigation, as the number of hypotheses tested by Mendelian randomization was not pre-specified in the analysis plan. We have therefore not accounted for multiple testing in our analyses, and so advise readers to interpret findings with additional caution.

Bayesian model averaging was performed by running the MR-BMA method using default settings, including an independent prior inclusion probability of 0.1 for each risk factor [24]. Bayesian model selection was performed by running the MVMR-cML-SuSiE method using default settings, except with initial variant pruning at a threshold of 0.9 [25]. The trait correlation matrix was calculated as the correlation of beta-coefficients.

All analyses were performed in the R software environment (version 4.0.2), using packages susieR (version 0.12.35), coloc (version 5.2.3), colocPropTest (version 0.9.1), prop.coloc (version 1.1.0), MendelianRandomization (version 0.9.0), mrbma (version 0.1.0), and MVMRcMLSuSiE (version 0.1.0). Further details describing each method are provided in the Supplementary Methods.

## Results

### Colocalization to identify phenotypic heterogeneity

**Figure 2.**
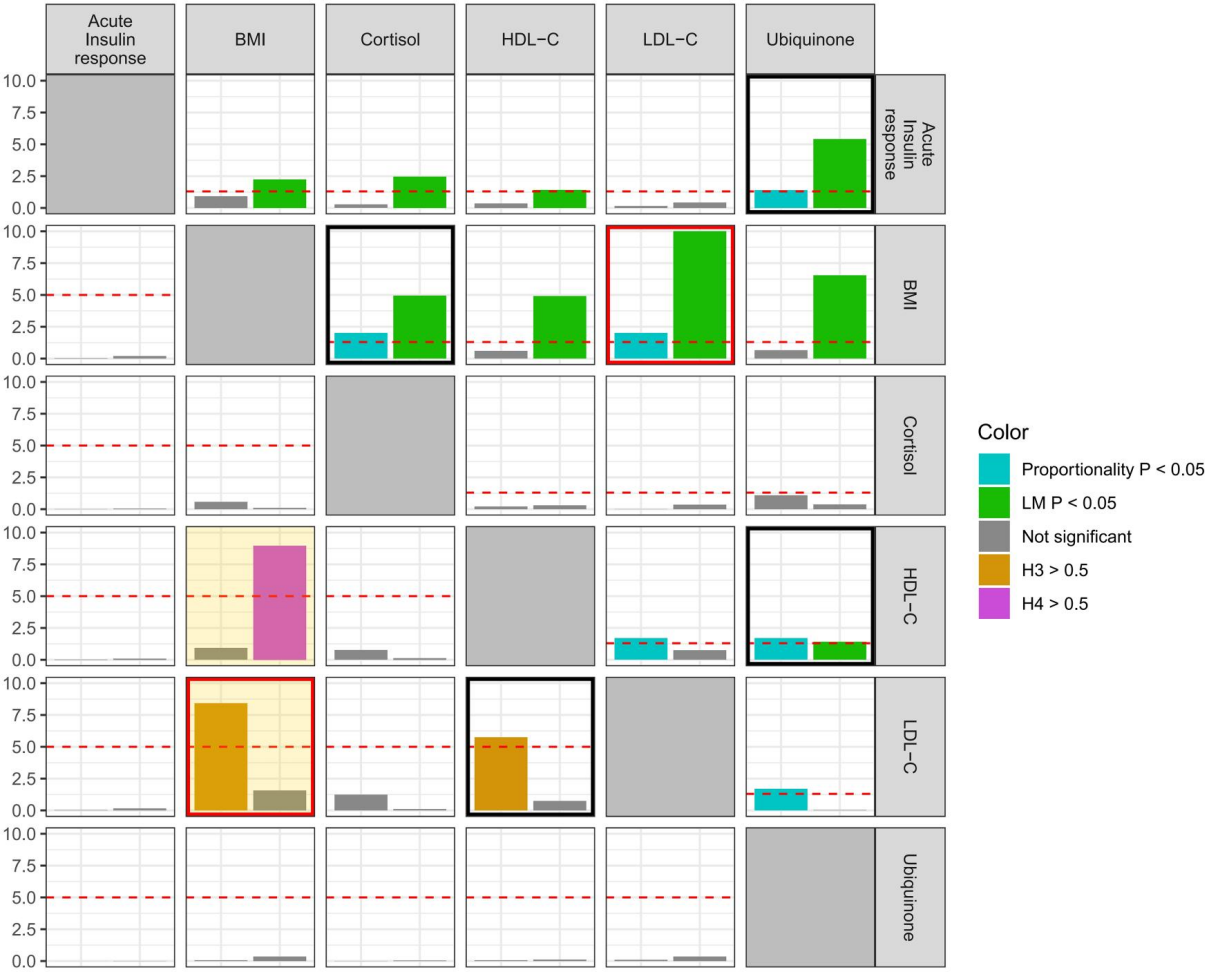
Heatmap of colocalization results from proportional colocalization and coloc-SuSiE/coloc methods. Top-right quadrant displays results from the proportional colocalization method (prop-coloc-cond). Bars represent negative log_10_-transformed *P* values for the proportionality test (cyan) and the Lagrange multiplier (LM) test (green). Phenotypic heterogeneity is indicated when both tests reject the null hypothesis (*P* < .05, equivalent to −log_10_  *P* > 1.3, red horizontal line). Bottom-left quadrant displays results from coloc-SuSiE (orange-shaded box) or coloc methods. Coloc-SuSiE was used preferentially; coloc was used in cases where coloc-SuSiE did not find a credible set of causal variants. Bars represent posterior probability percentage divided by 10 (i.e. 10.0 represents 100%) for non-colocalization (H3, orange) or colocalization (H4, magenta). Phenotypic heterogeneity is indicated when the posterior probability for H3 is above 50% (red horizontal line). A black box indicates evidence for phenotypic heterogeneity from each method, and a red box indicates evidence from both methods. Only traits with evidence of rejecting at least one colocalization test are displayed.

Results from analyses of pairs of risk factors for the proportional colocalization and coloc-SuSiE/coloc methods are displayed in Fig. 2. We show results for each risk factor that rejected at least one proportional colocalization test. We used the coloc-SuSiE method preferentially, and coloc in cases where coloc-SuSiE did not find a credible set of causal variants. Evidence for phenotypic heterogeneity was inferred from rejection (*P* < .05) of the proportionality and LM tests in the proportional colocalization method, and a high value (>50%) for the non-colocalization hypothesis (H3) in coloc-SuSiE or coloc. This was only observed for one pair of traits: LDL-C and BMI. Results from all credible sets using coloc-SuSiE between LDL-C and BMI are reported in Supplementary Table S2; for each pair of credible sets, the posterior probability of H3 (PP-H3) was >0.84.

To validate this further, we conducted the colocPropTest method, which also indicated non-proportionality in the genetic associations with BMI and LDL-C (Supplementary Fig. S1). We therefore focus on this pair in multivariable Mendelian randomization analyses. We note that this pair comprises one lipid trait and one metabolic trait.

### Multivariable Mendelian randomization for the *HMGCR* gene region

**Figure 3.**
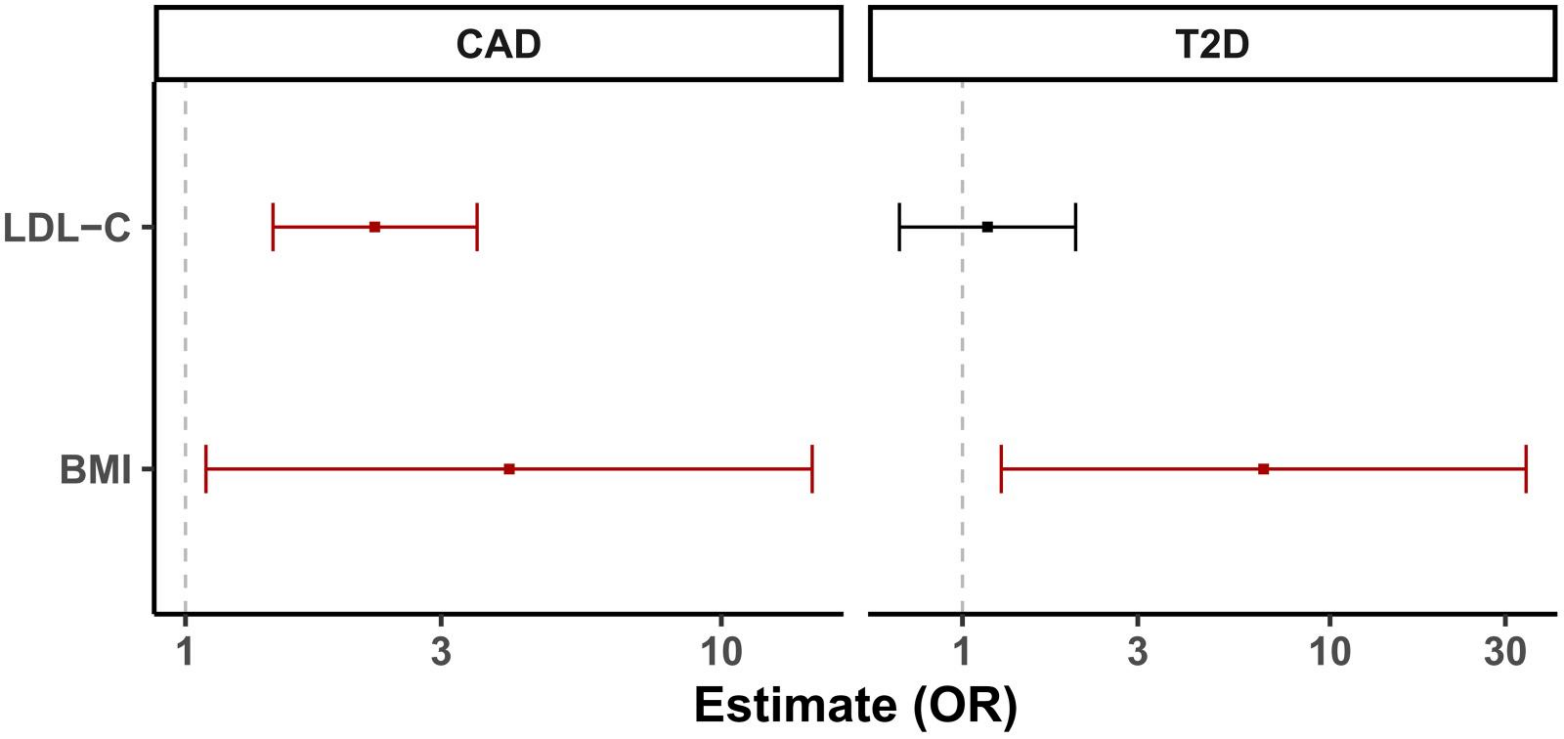
Multivariable Mendelian randomization results. Estimates represent mutually adjusted odds ratios for coronary artery disease (CAD, left) and type 2 diabetes (T2D, right) per 1 standard deviation increase in genetically predicted low-density lipoprotein cholesterol (LDL-C) or per 1 kg/m^2^ increase in body mass index (BMI) from multivariable analyses restricted to genetic variants in the *HMGCR* locus.

*cis*-Multivariable Mendelian randomization results are displayed in Fig. 3. Mutually adjusted genetically predicted levels of LDL-C and BMI were each independently associated with CAD risk: odds ratio (OR) 2.26 (95% confidence interval [CI]: 1.46, 3.50; *P* = .0003) per 1 standard deviation increase in LDL-C, and OR 4.02 (95% CI: 1.09, 14.78; *P* = .04) per 1 kg/m^2^ increase in BMI. In contrast, genetically predicted levels of BMI were conditionally associated with T2D risk: OR 6.60 (95% CI: 1.28, 34.16; *P* = .03), but there was no clear association for genetically predicted levels of LDL-C: OR 1.17 (95% CI: 0.67, 2.03; *P* = 0.58). Analyses were based on 119 variants for CAD and 96 variants for T2D; in both cases, the top 3 principal components explained 99% variance in the gene-exposure association matrix. Conditional *F*-statistics were 10.6 for LDL-C and 10.6 for BMI on CAD, and 11.1 for LDL-C and 11.0 for BMI on T2D. This indicates that the statistical power to detect effects of LDL-C and BMI should be similar, and there is not too much collinearity between the genetic associations with LDL-C and BMI. Confidence intervals are wider for T2D than for CAD due to greater overdispersion in the associations with the outcome. The magnitude of estimates is somewhat arbitrary, as (for example) the estimates could be doubled if we instead considered the odds ratio per 2 kg/m^2^ increase in BMI. Similar results were obtained in sensitivity analyses using different numbers of principal components (Supplementary Table S3).

### Colocalization to validate Mendelian randomization findings

We performed colocalization analyses to support or refute our Mendelian randomization findings. If an exposure colocalizes with an outcome, this increases our confidence that the exposure and outcome are on the same causal pathway. Results are summarized in Table 1, and full results from coloc-SuSiE when there were multiple credible sets are provided in Supplementary Table S4.

**Table 1.** Summary of results from colocalization analyses to validate Mendelian randomization.

		coloc/coloc-SuSiE		prop.coloc		colocPropTest
Method		H3	H4	Proportionality test	LM test	Proportionality test
LDL-C	CAD	<0.01	1.00	0.18	<0.01	1.00
	T2D	0.92*	0.08*	0.60	<0.01	0.68
BMI	CAD	0.18	0.82	0.04	<0.01	0.01
	T2D	0.03	0.97	0.97	<0.01	1.00

With the coloc/coloc-SuSiE methods, coloc-SuSiE was performed preferentially, and coloc was performed if coloc-SuSiE failed to find a credible set for either of the traits. Results for coloc/coloc-SuSiE are posterior probabilities for non-colocalization (H3) and colocalization (H4). A star (*) indicates that coloc-SuSiE found multiple credible sets, and that the stated results are for the first pair of sets; full results are given in Supplementary Table S4. Results from the prop.coloc method are *P* values for the Lagrange multiplier (LM) test (a significant result indicates a non-zero proportionality constant) and the proportionality test (a significant result indicates non-proportional genetic associations, i.e. non-colocalization). Results from the colocPropTest method are false discovery rate corrected *P* values (a significant result indicates non-proportional genetic associations, i.e. non-colocalization).

For LDL-C and CAD, there was strong evidence for colocalization from the coloc method (posterior probability of H4 [PP-H4] = 1.00), and no evidence to reject colocalization in either of the proportional colocalization methods. For LDL-C and T2D, there was strong evidence for non-colocalization from the coloc-SuSiE method, with PP-H3 > 0.92 for all four pairs of credible sets. However, the proportional colocalization methods did not reject colocalization.

For BMI and CAD, there was mixed evidence, with coloc-SuSiE favoring colocalization (PP-H3 = 0.18, PP-H4 = 0.82), but the proportional colocalization methods both rejected colocalization. For BMI and T2D, there was strong evidence for colocalization from the coloc method (PP-H4 = 0.97), and no evidence to reject colocalization in either of the proportional colocalization methods.

In summary, we found consistent evidence in the *HMGCR* gene region to validate LDL-C as a causal risk factor for CAD, and BMI as a causal risk factor for T2D, and inconsistent evidence for BMI as a causal risk factor for CAD, and LDL-C as a causal risk factor for T2D. Together, our findings suggest that BMI raising variants in the *HMGCR* region increase T2D risk, whereas LDL-C lowering variants reduce CAD but do not affect T2D risk. This is consistent with the effects of statins on CAD and T2D being mediated by distinct molecular mechanisms.

### Bayesian model averaging

Results from the MR-BMA method are shown in Table 2. For CAD, the top-ranking model was the model containing ubiquinone as the single risk factor, and the top-ranking risk factor by marginal inclusion probability (MIP) was ubiquinone (MIP = 27.68%), followed by LDL-C (MIP = 25.04%). We note that reduced ubiquinone is a direct molecular consequence of HMGCR inhibition, and hence, this is likely not a competing risk factor to LDL-C, but an upstream trait on shared causal pathways. For T2D, the top-ranked model was BMI as a single risk factor, and BMI was the top-ranked risk factor (MIP = 54.64%).

**Table 2.** Top ranking models and risk factors from Mendelian randomization Bayesian model averaging (MR-BMA) method: risk factors showing phenotypic heterogeneity in Fig. 2 are used as inputs in this method.

Coronary artery disease		
**Rank**	**Top models (subsets of risk factors)**	**Posterior probability (%)**
1	Ubiquinone	19.11
2	Acute insulin response	15.52
3	Cortisol	14.24
4	LDL-C	9.07
5	HDL-C	7.42
**Rank**	**Top risk factors**	**Marginal inclusion probability (%)**
1	Ubiquinone	27.68
2	LDL-C	25.04
3	Acute insulin response	23.61
4	Cortisol	23.17
5	BMI	17.47
**Type 2 diabetes**		
**Rank**	**Top models (subsets of risk factors)**	**Posterior probability (%)**
1	BMI	34.65
2	HDL-C	19.94
3	Ubiquinone	5.05
4	Acute insulin response	4.82
5	BMI, HDL-C	4.81
**Rank**	**Top risk factors**	**Marginal inclusion probability (%)**
1	BMI	54.64
2	HDL-C	34.55
3	Ubiquinone	14.60
4	Acute insulin response	14.46
5	Cortisol	13.55

### Bayesian variable selection

Results from the MVMR-cML-SuSiE method are shown in Table 3. For CAD, the top-ranking risk factor by posterior inclusion probability (PIP) was LDL-C (PIP = 99.95%). Several other risk factors had PIP above 50%, including BMI (PIP = 57.87%). For T2D, the only risk factor with PIP above 50% was BMI (PIP = 99.80%).

**Table 3.** Top ranking risk factors from Bayesian variable selection method (MVMR-cML-SuSiE): risk factors showing phenotypic heterogeneity in Fig. 2 are used as inputs in this method.

Coronary artery disease		
**Rank**	**Top risk factors**	**Posterior inclusion probability (%)**
1	LDL-C	99.95
2	HDL-C	64.98
3	Ubiquinone	61.05
4	Cortisol	60.82
5	BMI	57.87
**Type 2 diabetes**		
**Rank**	**Top risk factors**	**Posterior inclusion probability (%)**
1	BMI	99.80
2	Cortisol	45.73
3	Acute insulin response	34.35
4	HDL-C	6.58
5	Ubiquinone	4.67

## Discussion

In this investigation, we performed various statistical analyses using genetic association data for variants in the *HMGCR* gene region to investigate causal pathways influencing CAD and T2D. Colocalization analyses indicated that there are distinct genetic predictors of LDL-C and BMI, a finding suggesting that these traits are influenced by separate causal pathways. A targeted multivariable Mendelian randomization analysis including these two traits gave differing results for the two diseases: for CAD, it suggested that LDL-C and BMI were causal mediators of disease risk, and for T2D, it suggested that BMI was a mediator and LDL-C was not. Multivariable analyses, including a wider range of risk factors, gave similar results: Bayesian model averaging prioritized LDL-C as the second most likely causal risk factor for CAD (behind ubiquinone), whereas it prioritized BMI as the most likely causal risk factor for T2D. As ubiquinone is a generic biomarker of HMGCR inhibition, it is perhaps less suitable for distinguishing the mechanism by which HMGCR affects outcomes. Bayesian model selection prioritized LDL-C as the most likely risk factor for CAD, and BMI as the sole likely risk factor for T2D. Colocalization analyses indicated consistent evidence for LDL-C as colocalizing with CAD, and BMI colocalizing with T2D. Although we would be cautious about an overly literal interpretation of these results, it is clear that the results are very different for the two diseases. Together, our results suggest that HMGCR inhibition by statins may affect distinct causal pathways to cause T2D and protect against CAD.

Our findings are consistent with previous results from both experimental and observational studies. Randomized clinical trials have shown that LDL-C lowering using statins reduces CAD risk [34]. Moreover, multiple *in vivo* and clinical studies have substantiated a causal relationship between obesity and T2D [35]. Randomized trials have also shown that the weight loss drug semaglutide lowers the risk of cardiovascular disease [36]. Aside from clinical evidence, several Mendelian randomization analyses have been performed to verify the causal link between LDL-C and risk of CAD [37], the causal link between BMI and T2D [38], and the causal link between BMI and CAD [39], including analyses based on variants in the *HMGCR* locus [19]. However, previous analyses have not investigated the causal pathways underlying these signals, particularly using multivariable methods that are able to distinguish between pathways.

It has been suggested that lipophilic statins may have stronger effects on T2D risk than hydrophilic statins. This differential effect may stem from the ability of lipophilic statins to more efficiently cross the membranes of non-hepatic cells, such as pancreatic β-cells, adipocytes, and skeletal muscle cells, thereby influencing glucose metabolism and insulin secretion [40]. A network meta-analysis of clinical trials gave estimates for various statins on T2D risk [41]. For lipophilic statins, estimates were OR 1.34 (95% CI 1.14, 1.57) and 1.21 (95% CI 0.99, 1.49) for high-dose atorvastatin and simvastatin, and OR 1.13 (95% CI 0.94, 1.34) and 1.13 (95% CI 0.99, 1.29) for low-dose atorvastatin and simvastatin. For hydrophilic statins, estimates were OR 1.04 (95% CI 0.93, 1.16) and 1.17 (95% CI 1.02, 1.35) for pravastatin and rosuvastatin. Estimates are somewhat larger for lipophilic statins, although a greater difference is seen for high-dose versus low-dose statins. A difference between the magnitude of effect on T2D risk depending on hydrophobicity, and therefore possibly differential tissue actions, would support the broad conclusion of this work: namely, that there are distinct effects of HMGCR inhibition on T2D risk depending on the intervention, although further work would be needed to assess the tissue-specific contributions of statin-induced diabetes [42].

While statins have clear benefits in terms of reducing CAD risk, they have unwanted effects in terms of increasing T2D risk. Our results suggest that these effects are on different causal pathways, raising the possibility that targeted treatments could be developed to inhibit *HMGCR* in a more specific way that lowers CAD risk without increasing T2D risk. We attempted to provide greater insight into the mechanisms of action using data on *HMGCR* gene expression, but we did not find strong evidence for colocalization between gene expression and known downstream consequences of statins, such as LDL-C and BMI. Other investigators considering genetic proxies of drug targets have also found that gene expression is not always a reliable guide to the mechanism of action [43], and a recent review recommended using downstream traits to calibrate and guide choice of genetic variants where possible, rather than gene expression or protein levels [44]. However, while our analyses are not able to pinpoint how this could be achieved, it should encourage drug manufacturers to consider different mechanisms of action and modalities of treatment in drugs that target the mevalonate pathway, and that deeper functional characterization of these genetic effects is warranted. It also suggests that increases in T2D risk may not be seen equally for all LDL-C lowering targets. More generally, our investigation provides an example of how drug target Mendelian randomization can provide translational insights into drug development.

Our investigation has limitations. As with all Mendelian randomization analyses, we rely on the validity of the genetic variants as instrumental variables. More specifically, we rely on the genetic variants as satisfying the gene—environment equivalence assumption for statins [45]; i.e. the genetic variants influence traits and outcomes in a similar way to statins. This would not be satisfied if genetic variants have pleiotropic effects on other traits, or associations with other traits arising from linkage disequilibrium with a variant in another gene region, or from population stratification. We have minimized the possibility of this by restricting our analyses to variants in a narrow region around the *HMGCR* gene; however, we cannot fully discount this possibility. Our estimates may suffer from weak instrument bias. Weak instrument bias is particularly pervasive in multivariable Mendelian randomization, as we require not only strong genetic predictors of all the traits in the model, but also some degree of independence in these genetic predictors [46]. While the generalized method of moments is less sensitive to weak instruments than some other methods [47], some bias may remain. We chose a fairly stringent definition for phenotypic heterogeneity to minimize both bias from weak instruments and the multiple testing burden. Although genetic variants mimic interventions on drug targets in many aspects, genetic associations represent lifelong, small differences in risk factor levels, whereas pharmacological interventions are typically shorter in duration, but greater in magnitude [48]. We caution that exposures should be viewed as biomarkers for the effect of HMGCR inhibition. As such, our estimates do not represent the effects of the exposures on the outcomes, but rather the effect of HMGCR inhibition on the outcome as calibrated by the exposures. This is particularly true for BMI, as this does not represent the main effect of HMGCR inhibition, and so our estimates for the effects of BMI are larger than those from conventional Mendelian randomization analyses. Additionally, it is possible that the estimate for BMI reflects some unmeasured risk factor, such as the effect of cholesterol production in a tissue that is not reflected by blood cholesterol measures. Finally, our analyses were performed in predominantly European ancestry populations. While we would not expect strong differences in biological effects between ancestry groups, there are differences between populations in terms of disease prevalence, risk factor distributions, and response to metabolic changes [49] that could lead to different findings for different ancestry groups. We attempted to reproduce this analysis in BioBank Japan [50]. However, in this dataset, there was strong evidence for colocalization between LDL-C and BMI (97% posterior probability using coloc) based on the available genetic variants, and so we were unable to distinguish between these traits as separate risk factors using this dataset. There is no evidence that the effects of statins operate via different mechanisms in the BioBank Japan dataset. The reason for the discrepancy is not clear, but it may be due to different genetic architecture (i.e. allele frequencies and patterns of linkage disequilibrium) in the two ancestral populations.

In conclusion, we have found evidence from human genetics that different consequences of statin usage are on different causal pathways, and hence could be influenced separately by targeted interventions. Future investigations into drugs that inhibit the *HMGCR* pathway should investigate whether lipid lowering can be achieved without metabolic dysfunction.

## Ethics approval

Our study only uses publicly available summarized data, and so does not require specific ethical approval. Ethical approval for the original studies can be found in the relevant references.

## Supplementary Material

dyaf223_Supplementary_Data

## Data Availability

All datasets used to obtain estimates of genetic associations with risk factors and outcomes are listed in Supplementary Table S1. Linkage disequilibrium matrix data estimated in UK Biobank participants can be downloaded at https://registry.opendata.aws/ukbb-ld.

## Code availability

R scripts to apply all methods used in this manuscript, including multivariable Mendelian randomization, colocalization, and Bayesian model averaging, are available at https://github.com/sw4rsw4r/HMGCR-Causality and R markdown to allow the reader to reproduce all results, figures, and tables is available at https://sw4rsw4r.github.io/HMGCR-Causality/HMGCR-Causality.html.
