# Supplementary material for: Human genetics suggests differing causal pathways from HMGCR inhibition to coronary artery disease and type 2 diabetes
Source: Int J Epidemiol. 2026 Jan 5;55(1):dyaf223. doi: 10.1093/ije/dyaf223 (PMC12766909; doi:10.1093/ije/dyaf223)
Supplement: dyaf223_Supplementary_Data [file dyaf223_supplementary_data.docx]

**SUPPLEMENTARY MATERIAL for *HMGCR* mechanisms**

Contents

[SUPPLEMENTARY METHODS 1](#_Toc216885066)

[Colocalization 1](#_Toc216885067)

[Multivariable Mendelian randomization 3](#_Toc216885068)

[Bayesian model averaging 4](#_Toc216885069)

[Bayesian model selection 4](#_Toc216885070)

[SUPPLEMENTARY DATA 6](#_Toc216885071)

[Figure S1. Heatmap of colocalization results from proportional colocalization and colocPropTest methods. 6](#_Toc216885072)

[Table S1. List of risk factors and outcomes 7](#_Toc216885073)

[Table S2. Full coloc-SuSiE results for LDL-C and BMI: numbers in bold are used for plotting in Figure 2. 8](#_Toc216885074)

[Table S3. Sensitivity analysis for multivariable Mendelian randomization investigation varying the number of principal components 9](#_Toc216885075)

[Table S4. Full coloc-SuSiE results for LDL-C and T2D 10](#_Toc216885076)

[References 10](#_Toc216885077)

# SUPPLEMENTARY METHODS

## Colocalization

Colocalization is a statistical method to distinguish between two scenarios at a given gene region for a pair of traits: 1) the traits have shared genetic predictors (known as colocalization, and consistent with the traits being on the same causal pathway), and 2) the traits have distinct genetic predictors (known as non-colocalization, and consistent with the traits being on different causal pathways).^1^ While in typical applications of Mendelian randomization, we want to see colocalization between the exposure and outcome, in this case we are interested in finding pairs of traits that have distinct genetic predictors because this enables multivariable Mendelian randomization analyses using the two traits as exposures; if two risk factors have distinct (i.e. not proportional) genetic predictors, a multivariable model can distinguish between their effects.

Colocalization analyses were performed using four methods, which address slightly different questions and/or make slightly different assumptions. The coloc method considers evidence for five hypotheses.^2^ The critical hypotheses for this work are H3 (distinct causal variants for each trait; that is, non-colocalization) and H4 (shared causal variant for both traits; that is, colocalization). Other hypotheses are that there is only a causal variant for trait 1 (H1), only for trait 2 (H2), or no causal variants for either of the traits (H0). The coloc-SuSiE (sum of single effects) method is an extension of the coloc method that allows each trait to have multiple causal variants, represented by distinct credible sets.^3^ It considers whether or not there is colocalization for each pair of detected credible sets using the same set of hypotheses as coloc.

The proportional colocalization method assesses whether genetic associations with one trait are proportional with those of the other trait or not.^4^ Proportionality suggests that the two traits are on the same causal pathway. The prop.coloc method reports two *P* values: the Lagrange multiplier (LM) test, which tests the null hypothesis that the proportionality constant is zero, and the proportionality test, which tests the null hypothesis that the genetic associations are proportional. Non-colocalization is concluded when the LM test and proportionality tests both reject the null hypothesis. If the LM test rejects the null but the proportionality test does not, then there is no evidence to reject the colocalization hypothesis.

The colocPropTest method also assesses proportionality in genetic associations.^5^ It does this by taking pairs of variants in turn, assessing proportionality in genetic associations using a heterogeneity test for that pair, and using false discovery rates to account for multiple testing. If there is sufficient evidence, the null hypothesis of colocalization is rejected in favour of the alternative hypothesis of non-colocalization.

Colocalization analyses were performed for two purposes: first, we investigate colocalization between exposures to find pairs of exposures with separate genetic predictors, and second, we investigate colocalization between exposures used in our multivariable Mendelian randomization analyses with disease outcomes, to validate their status as causal risk factors.

## Multivariable Mendelian randomization

Standard Mendelian randomization takes genetic predictors of a single exposure, and assesses whether genetic predictors of that exposure (or equivalently, genetically-predicted levels of the exposure) are associated with the outcome in a univariable regression model. Under the instrumental variable assumptions^6^, an association between genetically-predicted levels of the exposure and the outcome is indicative of a causal effect of the exposure on the outcome.

Multivariable Mendelian randomization considers multiple related exposures, and assesses whether genetically-predicted levels of each exposure are conditionally associated with the outcome in a multivariable regression model.^7^ The motivation is that it can be difficult to find genetic variants that associate uniquely with a single exposure. Multivariable Mendelian randomization allows genetic variants to associate with any or all of the exposures in the model. Under the multivariable instrumental variable assumptions, a conditional association between genetically-predicted levels of an exposure and the outcome is indicative of a direct causal effect of that exposure on the outcome.^8^

In our example, genetic variants in the *HMGCR* gene region are associated with multiple risk factors that can be used as separate exposures in multivariable Mendelian randomization provided that their genetic associations are not proportional (as this would lead to collinearity in the regression model). This method can identify the proximal causal risk factors for an outcome. We perform multivariable Mendelian randomization for pairs of risk factors that have strong evidence for having non-proportional genetic associations (i.e. evidence for non-colocalization in the framework above).^9^ We refer to risk factors with non-proportional genetic associations as displaying “phenotypic heterogeneity”.

## Bayesian model averaging

Rather than considering pairs of risk factors separately, the Mendelian randomization Bayesian model averaging (MR-BMA)^10^ method allows the consideration of all risk factors in a model averaging framework. We include all risk factors displaying some evidence of phenotypic heterogeneity in this analysis. We perform Mendelian randomization for each possible model (that is, each subset of risk factors): each risk factor alone, each pair of risk factors, each triple of risk factors, and so on. For a small number of risk factors, this process can consider all models; for a larger number of risk factors, a stochastic search strategy is deployed. Each set of risk factors is assigned a Bayes factor depending on how well the data fit the model, and these are used to calculate the posterior probability of each model (each subset of risk factors). We also calculate the marginal inclusion probability (MIP) for each risk factor, representing the sum of posterior probabilities for models containing that risk factor. This provides a ranking of risk factors based on their likelihood of being a causal risk factor for the outcome.

## Bayesian model selection

MVMR-cML-SuSiE is an extension of the MVMR framework that performs MVMR for large numbers of potentially highly genetically-correlated risk factors in a single model, and selects the most plausible risk factors to explain the genetic associations with the outcome.^11^ It leverages constrained maximum likelihood (cML) to model genetic associations while mitigating against bias from horizontal pleiotropy,^12^ and employs Bayesian model selection using the SuSiE algorithm to identify independent signal clusters (credible sets) of risk factors. The method then assigns a posterior inclusion probability (PIP) to each risk factor.

More specifically, the method: (i) works as a model selection procedure that jointly selects the most plausible risk factors; (ii) aims to find plausibly valid instruments for those plausible risk factors; and (iii) estimates the resulting MVMR model in a way that is robust to a subset of selected instruments being invalid. The key assumption underpinning the approach is that a plurality of selected instruments are valid instruments.

To select the most plausible risk factors, the method employs Bayesian model selection using the SuSiE algorithm which operates under a sparse effects assumption^13^. The algorithm groups potential risk factors into clusters, and assigns a PIP to each risk factor. Within each cluster, only those risk factors that achieve a certain PIP threshold are considered to be plausibly relevant. Enumerating all plausibly relevant risk factors across all clusters results in multiple plausible models. The MVMR-cML procedure (that aims to select plausibly valid instruments for a given model) is applied to each plausible model.

The key difference is that the MVMR-cML-SuSiE method models the direct pleiotropic variant effects on the outcome as "non-random"; the inclusion of such variants may induce a bias in the MR causal effect estimates. On the other hand, under a random-effects model, all variants are permitted to directly affect the outcome, such that the effects are “random” around zero with an unknown variance. The effect of such random pleiotropic effects may inflate the variance in MR causal effect estimates, rather than affect its bias, potentially leading to a more credible assessment of uncertainty in MR causal effect estimates. The key assumption of the MVMR-cML-SuSiE method is that a plurality of selected instruments are valid, whereas the key assumption of the random-effects model is that the direct pleiotropic effects are random around zero, and so all included instruments are permitted to be invalid in the random-effects approach, although more restrictively. Both approaches aim to deliver MVMR results that are robust to invalid instruments.

Use of this method was suggested by a reviewer; it was not part of the original statistical analysis plan.

# SUPPLEMENTARY DATA


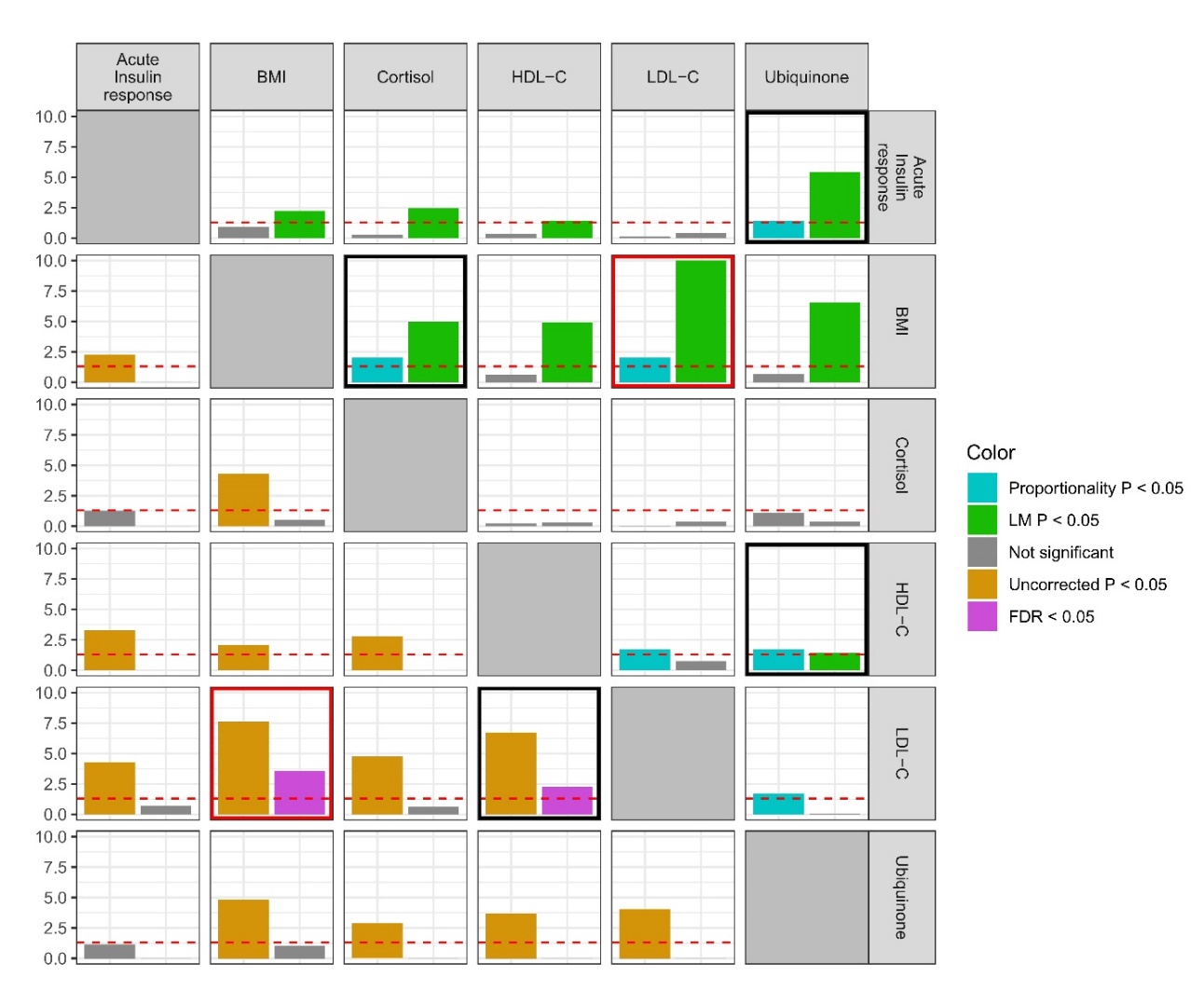


## Figure S1. Heatmap of colocalization results from proportional colocalization and colocPropTest methods.

Top-right quadrant displays results from proportional colocalization method (prop-coloc-cond). Bars represent negative log_10_-transformed *P* values for the proportionality test (cyan) and the Lagrange multiplier (LM) test (green). Phenotypic heterogeneity is indicated when both tests reject the null hypothesis (p<0.05, equivalent to ‑log_10_p>1.3, red horizontal line). Bottom-left quadrant displays results from colocPropTest method. Bars represent negative log_10_-transformed *P* values for the proportionality test: uncorrected *P* value (orange), and false-discovery rate (FDR) corrected *P* value (magenta). Phenotypic heterogeneity is indicated when the FDR corrected *P* value is less than 0.05 (horizontal red line). A black box indicates evidence for phenotypic heterogeneity from each method, a red box indicates evidence from both frameworks. Only traits with evidence of rejecting at least one colocalization test are displayed.

**ALT TEXT**: Pairwise plots showing colocalization results from proportional colocalization and colocPropTest methods, and showing phenotypic heterogeneity when there is consensus between these methods.

## Table S1. List of risk factors and outcomes

| Phenotype | Study reference | GWAS Catalog  dataset ID or weblink |
| --- | --- | --- |
| Acute insulin response | Wood et al, Diabetes 2017; 66:2296-2309 | GCST004575 |
| Fasting insulin | Manning et al, Nat Genet 2012; 44:659-669 | GCST005185 |
| Fasting glucose | Manning et al, Nat Genet 2012; 44:659-669 | GCST005186 |
| BMI | Pulit et al, Hum Mol Genet. 2019; 28:166-174 | GCST009004 |
| LDL-C | Graham et al, Nature 2021; 600:675-679 | GCST90239658 |
| HDL-C | Graham et al, Nature 2021; 600:675-679 | GCST90239652 |
| Triglycerides | Graham et al, Nature 2021; 600:675-679 | GCST90239664 |
| Leptin | Yaghootkar et al, Diabetes. 2020; 69:2806-2818 | GCST90007310 |
| Sterol | Harshfield et al, BMC Medicine 2021; 19:232 | GCST90060133 |
| Cortisol | Chen et al, Nat Genet 2023; 55:44-53 | GCST90200378 |
| Testosterone | Leinonen et al, Communications Medicine. 2023; 3:4 | GCST90239820 |
| Estradiol | Schmitz et al, J Clin Endocrinol Metab. 2021;106: e4471-e4486 | GCST90020092 |
| Vitamin D | Revez et al, Nat Commun 2020; 11:1647 | GCST90000618 |
| Bile acid | Harshfield et al, BMC Medicine 2021; 19:232 | GCST90060135 |
| Aldosterone | Dennis et al, Genome Med 2021; 13:6 | GCST90012609 |
| Ubiquinone | Cadby et al, Nat Commun 2022; 13:3124 | GCST90024608 |
| CAD | Aragam et al, Nat Genet. 2022;54: 1803–1815 | GCST90132314 |
| T2D | Mahajan et al, Nat Genet. 2022;54: 560–572 | [https://www.diagram-consortium.org/downloads.html](https://www.diagram-consortium.org/downloads.html%20%20) (13 December 2025, date last accessed) |

## Table S2. Full coloc-SuSiE results for LDL-C and BMI: numbers in bold are used for plotting in Figure 2.

| LDL-C and BMI | | | Posterior probability for hypothesis: | | | | |
| --- | --- | --- | --- | --- | --- | --- | --- |
| Number of SNPs | Lead SNP for LDL-C | Lead SNP for BMI | H0 | H1 | H2 | H3 | H4 |
| **148** | **rs12916** | **rs3843480** | **<0.01** | **<0.01** | **<0.01** | **0.84** | **0.16** |
| 148 | rs72633963 | rs3843480 | <0.01 | <0.01 | <0.01 | 1.00 | <0.01 |
| 148 | rs184049365 | rs3843480 | <0.01 | <0.01 | <0.01 | 1.00 | <0.01 |
| 148 | rs7717396 | rs3843480 | <0.01 | <0.01 | <0.01 | 1.00 | <0.01 |
| 148 | rs113586903 | rs3843480 | <0.01 | <0.01 | <0.01 | 1.00 | <0.01 |

There are five credible sets in coloc-SuSiE for LDL-C and one for BMI. Colocalization between each pair of sets indicated evidence for distinct causal variants (H3).

## Table S3. Sensitivity analysis for multivariable Mendelian randomization investigation varying the number of principal components

| Number of principal components | *P* value (CAD) | | Number of principal components | *P* value (T2D) | |
| --- | --- | --- | --- | --- | --- |
|  | BMI | LDL-C |  | BMI | LDL-C |
| 3 (99%) | 0.036 | <0.001 | 3 (99%) | 0.024 | 0.581 |
| 4 | 0.039 | <0.001 | 4 | 0.027 | 0.708 |
| 5 (99.9%) | 0.029 | <0.001 | 5 (99.9%) | 0.017 | 0.615 |
| 6 | 0.01 | <0.001 | 6 | 0.002 | 0.184 |
| 7 | 0.009 | <0.001 | 7 | 0.002 | 0.197 |
| 13 (99.99%) | 0.004 | <0.001 | 12 (99.99%) | 0.001 | 0.780 |

For each number of principal components, multivariable Mendelian randomization indicated a causal role of LDL-C and BMI for coronary artery disease (CAD), and a causal role of BMI (but not LDL-C) for type 2 diabetes (T2D).

## Table S4. Full coloc-SuSiE results for LDL-C and T2D

| LDL-C and T2D | | | Posterior probability for hypothesis: | | | | |
| --- | --- | --- | --- | --- | --- | --- | --- |
| Number of SNPs | Lead SNP for LDL-C | Lead SNP for T2D | H0 | H1 | H2 | H3 | H4 |
| 111 | rs12916 | rs7733436 | <0.01 | <0.01 | <0.01 | 0.92 | 0.08 |
| 111 | rs138091235 | rs7733436 | <0.01 | <0.01 | <0.01 | 1.00 | <0.01 |
| 111 | rs4703671 | rs7733436 | <0.01 | <0.01 | <0.01 | 0.98 | 0.01 |
| 111 | rs78585310 | rs7733436 | <0.01 | <0.01 | <0.01 | 0.99 | <0.01 |

There are four credible sets in coloc-SuSiE for LDL-C and one for T2D. Colocalization between each pair of sets indicated evidence for distinct causal variants (H3).

## References

1. Zuber V, Grinberg NF, Gill D, et al. Combining evidence from Mendelian randomization and colocalization: Review and comparison of approaches. *The American Journal of Human Genetics*. Elsevier; 2022;**109**(5):767–782.

2. Giambartolomei C, Vukcevic D, Schadt EE, et al. Bayesian test for colocalisation between pairs of genetic association studies using summary statistics. *PLoS Genet*. Public Library of Science San Francisco, USA; 2014;**10**(5):e1004383.

3. Wallace C. A more accurate method for colocalisation analysis allowing for multiple causal variants. *PLoS Genet*. Public Library of Science San Francisco, CA USA; 2021;**17**(9):e1009440.

4. Patel A, Whittaker JC, Burgess S. A frequentist test of proportional colocalization after selecting relevant genetic variants. *arXiv preprint arXiv:240212171*. 2024;

5. Chris Wallace. colocPropTest: Proportional Testing for Colocalisation Analysis. 2024.

6. Emdin CA, Khera A V, Kathiresan S. Mendelian randomization. *JAMA*. American Medical Association; 2017;**318**(19):1925–1926.

7. Sanderson E, Davey Smith G, Windmeijer F, Bowden J. An examination of multivariable Mendelian randomization in the single-sample and two-sample summary data settings. *Int J Epidemiol*. Oxford University Press; 2019;**48**(3):713–727.

8. Sanderson E. Multivariable Mendelian randomization and mediation. *Cold Spring Harb Perspect Med*. Cold Spring Harbor Laboratory Press; 2021;**11**(2):a038984.

9. Patel A, Gill D, Shungin D, et al. Robust use of phenotypic heterogeneity at drug target genes for mechanistic insights: Application of cis‐multivariable Mendelian randomization to GLP1R gene region. *Genet Epidemiol*. Wiley Online Library; 2024;**48**(4):151–163.

10. Zuber V, Colijn JM, Klaver C, Burgess S. Selecting likely causal risk factors from high-throughput experiments using multivariable Mendelian randomization. *Nat Commun*. Nature Publishing Group UK London; 2020;**11**(1):29.

11. Chan LS, Malakhov MM, Pan W. A novel multivariable Mendelian randomization framework to disentangle highly correlated exposures with application to metabolomics. *The American Journal of Human Genetics*. Elsevier; 2024;**111**(9):1834–1847.

12. Lin Z, Xue H, Pan W. Robust multivariable Mendelian randomization based on constrained maximum likelihood. *The American Journal of Human Genetics*. Elsevier; 2023;**110**(4):592–605.

13. Wang G, Sarkar A, Carbonetto P, Stephens M. A simple new approach to variable selection in regression, with application to genetic fine mapping. *J R Stat Soc Series B Stat Methodol*. Oxford University Press; 2020;**82**(5):1273–1300.
